# Multigene Phylogeny Reveals *Haploanthostomella elaeidis* gen. et sp. nov. and Familial Replacement of *Endocalyx* (Xylariales, Sordariomycetes, Ascomycota)

**DOI:** 10.3390/life11060486

**Published:** 2021-05-26

**Authors:** Sirinapa Konta, Kevin D. Hyde, Prapassorn D. Eungwanichayapant, Samantha C. Karunarathna, Milan C. Samarakoon, Jianchu Xu, Lucas A. P. Dauner, Sasith Tharanga Aluthwattha, Saisamorn Lumyong, Saowaluck Tibpromma

**Affiliations:** 1 CAS Key Laboratory for Plant Diversity and Biogeography of East Asia, Kunming Institute of Botany, Chinese Academy of Sciences, Kunming 650201, China; sirinapakonta@gmail.com (S.K.); samanthakarunarathna@gmail.com (S.C.K.); jxu@mail.kib.ac.cn (J.X.); luke.dauner1@gmail.com (L.A.P.D.); 2 Center of Excellence in Fungal Research, Mae Fah Luang University, Chiang Rai 57100, Thailand; kdhyde3@gmail.com (K.D.H.); milan.chameera@yahoo.com (M.C.S.); 3 School of Science, Mae Fah Luang University, Chiang Rai 57100, Thailand; prapassorn@mfu.ac.th; 4 World Agroforestry Centre, East and Central Asia, Kunming 650201, China; 5 Centre for Mountain Futures, Kunming Institute of Botany, Kunming 650201, China; 6 Guangxi Key Laboratory of Forest Ecology and Conservation, College of Forestry, Guangxi University, Daxuedonglu 100, Nanning 530004, China; aluthwattha@yahoo.com; 7 State Key Laboratory of Conservation and Utilization of Subtropical Agro-Bioresources, College of Forestry, Guangxi University, Daxuedonglu 100, Nanning 530004, China; 8 Research Center of Microbial Diversity and Sustainable Utilization, Faculty of Science, Chiang Mai University, Chiang Mai 50200, Thailand; saisamorn.l@cmu.ac.th; 9 Academy of Science, The Royal Society of Thailand, Bangkok 10300, Thailand

**Keywords:** Apiosporaceae, Cainiaceae, fungi, palms, Thailand, Xylariales

## Abstract

During our investigation of palm fungi in Thailand, two interesting taxa from *Elaeis guineensis* and *Metroxylon sagu* (Arecaceae) were collected. Based on phylogenetic analyses of a combined dataset of ITS, LSU, *rpb2*, and *tub2* nucleotide sequences as well as unique morphological characteristics, we introduce the new genus *Haploanthostomella* within Xylariales, and a new species *Endocalyx metroxyli*. Additionally, in our study, the genus *Endocalyx* is transferred to the family Cainiaceae based on its brown conidia and molecular phylogenetic evidence.

## 1. Introduction

Palm trees represent a family of perennial lianas and consist of many diverse species worldwide, with the fossil record indicating around 65 million years of evolutionary history [1]. Microfungi on palms have been studied, but only a few have been analyzed using morphology and DNA sequence data. Several fungal species are currently unknown to science, with the total number estimated at somewhere between 2.2 and 3.8 million [2]. Thus, palms are a particularly interesting plant family for studying microfungi species unknown to science.

The subclass Xylariomycetidae has recently been updated to contain three orders (Amphisphaeriales, Delonicicolales, and Xylariales) and 35 families [3]. Recently, the family Induratiaceae was introduced in this subclass by Samarakoon et al. [4] with an updated phylogeny of Xylariales. Cainiaceae is a family of particular interest, as all members in this family tend to be found on monocotyledons, the majority of which are grasses [5]. In previous studies, Cainiaceae was accepted in the Xylariales [3,6]. Later, Hongsanan et al. [7], and Wijayawardene et al. [8] assigned Cainiaceae to the Xylariomycetidae as an *incertae sedis* family.

The Xylariales is one of the largest orders and includes 15 families, 160 genera, and 52 genera *incertae sedis* [3]. Family Cainiaceae was introduced by Krug [9] to include species of *Cainia* with unique apical rings in the asci that consist of a series of rings and ascospores with longitudinal germ slits. An asexual morph of Cainiaceae was coelomycetous with black, scattered, immersed pycnidial conidiomata; hyaline, denticulate, sympodially proliferating conidiophores; hyaline, filiform, branched or simple, septate conidiogenous cells with one to three phialides; and hyaline, elongate fusiform, falcate to lunate, unicellular or septate conidia, with pointed ends [10]. At present, seven genera have been accepted into this family (*Alishanica*, *Amphibambusa*, *Arecophila*, *Atrotorquata*, *Cainia*, *Longiappendispora*, and *Seynesia*) [3,11].

Since 2014, fungal research in Thailand has revealed a high diversity of novel species [12,13,14]. In this study, we found fungal species unknown to science from Thailand. The phylogeny results show that *Endocalyx* grouped within Cainiaceae, and so we transferred *Endocalyx* from Apiosporaceae (Amphisphaeriales) to Cainiaceae (Xylariales) based on both morphology and multigene phylogeny. We also introduce the new species *Endocalyx metroxyli*, collected from the economically important oil palm host (*Elaeis guineensis*). Lastly, we introduce the new genus *Haploanthostomella* associated with true sago palm (*Metroxylon sagu*).

## 2. Materials and Methods

### 2.1. Collection, Isolation, and Identification

Saprobic fungi growing on dead leaves, petioles and rachis of *Elaeis guineensis* and *Metroxylon sagu* were collected in Krabi and Surat Thani Provinces of Thailand, placed in ziplock bags and brought to the mycology laboratory at the Center of Excellence in Fungal Research, and morphological characteristics were observed. Specimens were examined following the methods provided by Konta et al. [15]. Single spore isolates were obtained following the method of Senanayake et al. [16], using malt extract agar (MEA) and incubating at 25–28 °C overnight. Germinating conidia were transferred to new MEA media and pure cultures were kept at 25–28 °C. Specimens and cultures were deposited in the herbarium of Mae Fah Luang University (MFLU) and Mae Fah Luang University Culture Collection (MFLUCC), Chiang Rai, Thailand, respectively. Faces of Fungi and Index Fungorum numbers were registered as outlined in Jayasiri et al. [17] and Index Fungorum [18].

### 2.2. DNA Extraction and Amplification (PCR)

Genomic DNA was extracted from fruiting bodies of *Haploanthostomella elaeidis* and fungal mycelium of *Endocalyx metroxyli*. DNA extraction and amplification were followed Dissanayake et al. [19]. Konta et al.’s method [16] was followed for PCR amplification of ITS, LSU, SSU, *tef1-α* and *rpb2*, while O’Donnell and Cigelnik’s method [20] was followed for PCR amplification of the *tub2* region. Amplification was done using the primers ITS5 and ITS4 for the internal transcribed spacer regions and intervening 5.8S rDNA (ITS), the primers LR5 and LR0R for the large subunit (LSU) rRNA gene, the primer pair fRPB2-5f and fRPB2-7cR for the RNA polymerase II second largest subunit (*rpb2*) gene, and the primers T1 and T22 for the partial gene β-tubulin (*tub2*). PCR amplifications were performed using 1× PCR buffer with 8.5 μL ddH_2_O, 12.5 μL 2× Easy Taq PCR SuperMix (mixture of Easy Taq TM DNA Polymerase, dNTPs and optimized buffer (Beijing Trans Gen Biotech Co., Beijing, China)), 2 μL of DNA template, and 1 μL each of forward and reverse primers (10 pM) in a final volume of 25 μL. The cycle conditions in the initiation step were started at 95 °C for 3 min, followed by 35 cycles at 95 °C for 30 s, 55 °C for 50 s, 72 °C for 30 s (for ITS, LSU); 95 °C for 5 min, followed by 35 cycles at 95 °C for 1 min, 54 °C for 2 min, 72 °C for 1:5 min (for *rpb2*); 95 °C for 5 min, followed by 35 cycles at 94 °C for 1 min, 52 °C for 1 min, 72 °C for 1:5 min (for *tub2*); a final elongation step at 72 °C for 10 min and a final hold at 4 °C were done as the last steps. Purification and sequencing were performed by Sangon Biotech Co., Shanghai, China. Consensus sequences were computed using SeqMan software, and new sequences generated in this study were deposited in GenBank (Table 1).

### 2.3. Phylogenetic Analyses

The consensus sequences were put through a BLAST search in the NCBI GenBank nucleotide database to search for the fungal sequences of closest relatives that have been deposited in the NCBI database. Dissanayake et al.’s study [19] was followed for the phylogenetic analyses. Voglmayr and Beenken’s study [79] was used as a reference of the dataset. Both individual and combined ITS, LSU, *rpb2*, and *tub2* nucleotide sequences were analyzed. A total of 151 taxa were used for the phylogenetic analyses in order to find the taxonomic placement of each species. Three genera viz. *Delonicicola*, *Furfurella* (Delonicicolaceae), and *Leptosillia* (Leptosilliaceae) in Delonicicolales were used as the outgroup taxa.

**Figure 1:**
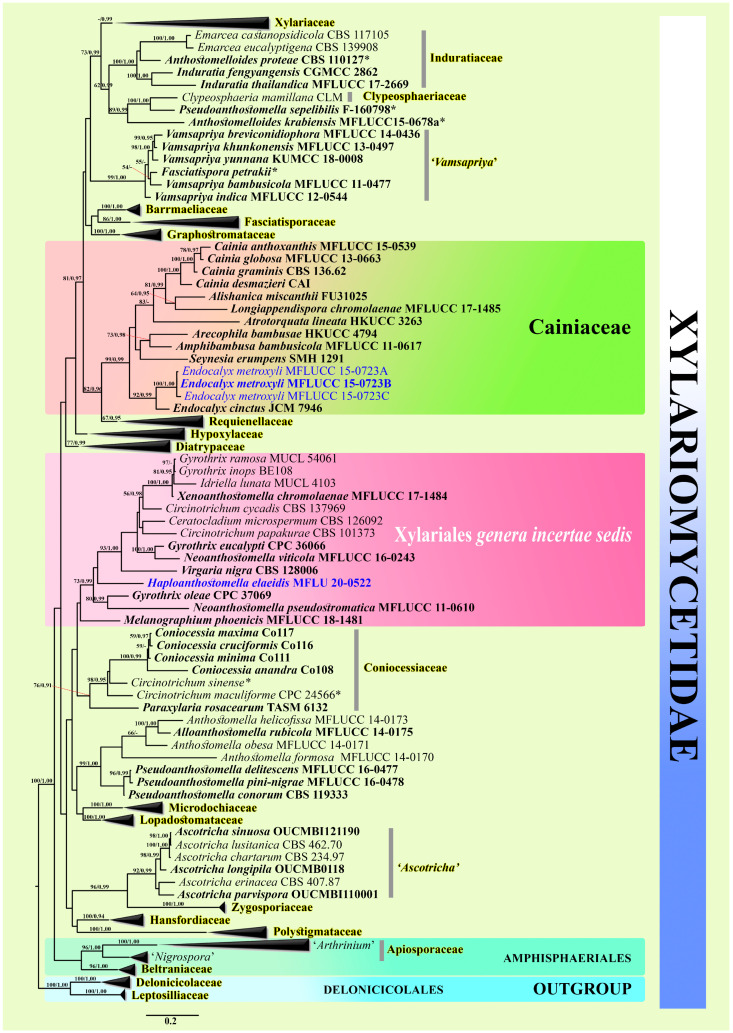
Maximum likelihood majority rule consensus tree for the analyses of selected Xylariomycetidae isolates based on a dataset of combined ITS, LSU, *rpb2*, and *tub2* nucleotide sequence. Bootstrap support values for maximum likelihood (ML) equal to or higher than 50% are given above each branch. Bayesian posterior probabilities (BYPP) equal to or greater than 0.90 are given at the nodes. Novel taxa are in blue bold and ex-type strains are in black bold. The tree is rooted to Delonicicolaceae and Leptosilliaceae (Delonicicolales). The asterisks represent unstable species.

The MAFFT online program was used to obtain initial alignments for each locus [94]. Alignments were manually edited and single gene sequence data sets were combined using MEGA7 [95]. The Alignment Transformation Environment online program was used to convert the file format [96]. MrModeltest [97] was used to find the best model for maximum likelihood (ML) and Bayesian analyses (BYPP). The six simultaneous Markov chains were run for 20,000,000 generations and trees were sampled every 1000th generation. Bayesian posterior probabilities from MCMC were evaluated with a final average standard deviation of the split frequency of <0.01. Bootstrap values for ML equal to or greater than 50% and BYPP equal to or greater than 0.90 are given at the nodes (Figure 1). Fig Tree v1.4.0 was used to configure the phylogenetic trees [98] and edited using Microsoft Office PowerPoint 2010 and Adobe Photoshop CS6 (Adobe Systems Incorporated, 345 Park Avenue, San Jose, CA, USA).

## 3. Results

### 3.1. Morphology and Phylogeny

The combined dataset comprised 151 taxa from selected taxa in Amphisphaeriales, Delonicicolales, and Xylariales (Table 1). The RAxML analyses of the combined dataset yielded the best-scoring tree (Figure 1) with a final ML optimization likelihood value of −126584.196783. The matrix had 4598 distinct alignment patterns, with 65.07% undetermined characters or gaps. Estimated base frequencies were: A = 0.243574, C = 0.257762, G = 0.258457, T = 0.240207; substitution rates AC = 1.296272, AG = 3.089851, AT = 1.400263, CG = 1.060328, CT = 9.900102, GT = 1.000000; gamma distribution shape parameter α = 0.443932. Tree-Length = 25.372161. Bayesian analysis resulted in a tree with similar topology and clades as the ML tree. Phylogenetic analyses of the combined ITS, LSU, *rpb2*, and *tub2* loci show two novel taxa within the monospecific genus *Haploanthostomella* (type species *Haploanthostomella elaeidis*; Xylariales *incertae sedis*) and the novel taxa *Endocalyx metroxyli*, with the genus *Endocalyx* being placed in Cainiaceae.

#### 3.1.1. ***Haploanthostomella*** Konta & K.D. Hyde. gen. nov.

Index Fungorum number: IF557876; Facesoffungi number: FoF09173

Etymology: “*haplos*” (απλός) in Greek means single; *Anthostomella* refers to its morphological similarity to *Anthostomella*.

*Saprobic* on dead leaves and rachis in terrestrial habitats. Sexual morph: *Ascomata* immersed in the host epidermis, beneath a clypeus, visible as slightly raised blackened areas, dark brown to black, coriaceous, solitary or aggregated into clusters, scattered, with an ostiolar canal. *Peridial wall* thick, comprised of several layers of cells, outwardly comprising dark brown cells of *textura prismatica* and inwardly comprising hyaline cells of *textura angularis*. *Paraphyses* septate, tapering hyphae-like, hyaline. *Asci* eight-spored, unitunicate, clavate to cylindric, short pedicellate, with J-, apical ring. *Ascospores* uni–biseriate into the asci, unicellular, obovoid, fusoid, hyaline or brown to dark brown, verrucose with a mucilaginous cap at apex. *Germ slit* straight, less than spore-length. Asexual morph: Not observed.

Type species: *Haploanthostomella elaeidis* Konta & K.D. Hyde.

Notes: *Anthostomella* species were proven to be polyphyletic, and it is of no surprise that a new genus with anthostomella-like characteristics was discovered in this study [99]. Phylogenetic analyses based on a single dataset of ITS (supporting information section) and combined sequence data indicated that *Haploanthostomella* belongs to Xylariales genera *incertae sedis*, separating well from other genera but with low bootstrap values (Figure 1). According to the phylogenetic tree (Figure 1), seven genera (*Ceratocladium*, *Circinotrichum*, *Gyrothrix*, *Idriella*, *Neoanthostomella*, *Virgaria* and *Xenoanthostomella*) are closely related to our new genus, but morphological characteristics of these genera are different. The genera *Neoanthostomella*, *Virgaria*, and *Xenoanthostomella* were compared morphologically since they are similar to our new taxon. *Haploanthostomella* differs from *Virgaria*, *Neoanthostomella*, and *Xenoanthostomella* in having a J- apical ring, fusoid-obovoid ascospores, and verrucose with a mucilaginous cap at the apex, while *Virgaria* has asci with a J+ apical ring and smooth-walled elliposidal ascospores lacking of a mucilaginous sheath; *Neoanthostomella* smooth-walled elliposidal ascospores surrounded by a thick mucilaginous sheath; *Xenoanthostomella* has unilocular ascoma, and ascospores lacking germ slits and mucilaginous sheaths [13,72,89]. Therefore, *Haploanthostomella* is described here as a new genus based on phylogeny coupled with morphology. In addition, we provide a key to genera with *Anthostomella*-like characteristics.

#### 3.1.2. ***Haploanthostomella elaeidis*** Konta & K.D. Hyde., sp. nov.

**Figure 2:**
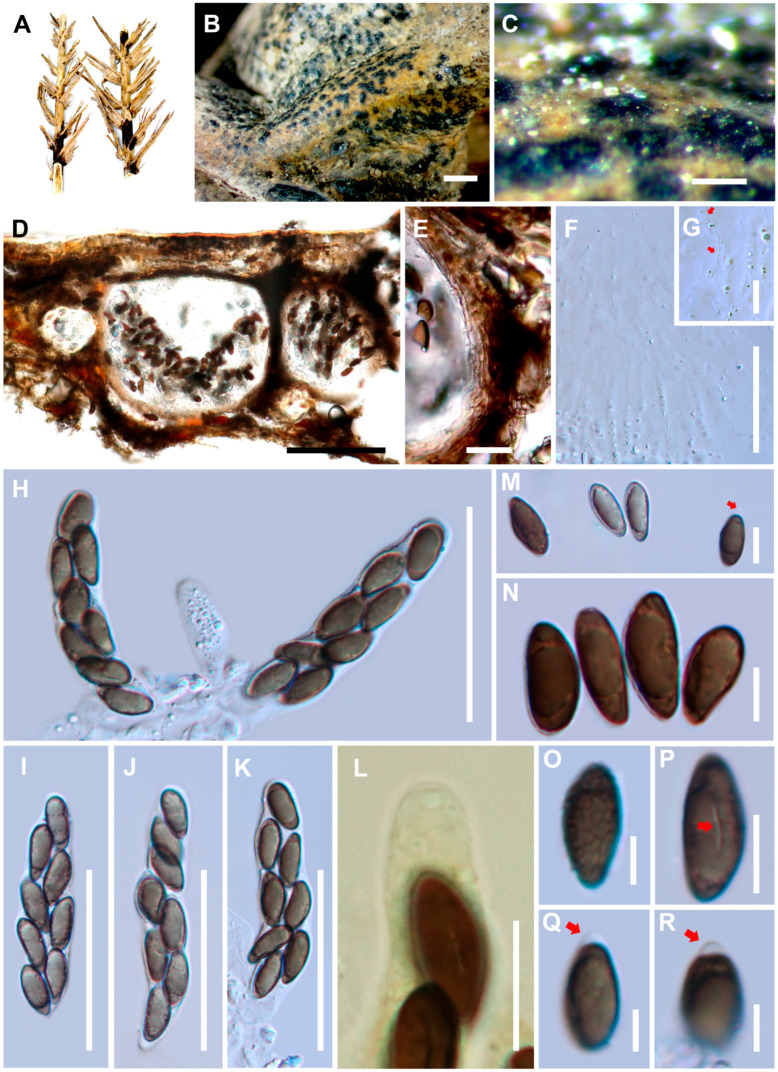
*Haploanthostomella elaeidis* (MFLU 20-0522, holotype). (**A**) Substrate. (**B**,**C**) Appearance of ascomata on the host surface. (**D**) Sections of ascomata. (**E**) Peridium. (**F**) Hamathecium. (**G**) Septa of paraphyses show in red arrows. (**H**,**I**–**K**) Asci. (**L**) J- apical ring in Melzer’s reagent. (**M**,**N**,**P**–**R**) Ascospores with mucilaginous cap (red arrows in M, Q, R) and germ slit (red arrows in P). (**O**) An ascospore with verrucose wall. Scale bars: B = 1000 μm, C = 200 μm, D = 500 μm, E, G, L = 20 μm, F, H–K = 50 μm, M–P = 10 μm, Q–R = 5 μm.

Index Fungorum number: IF557877, Facesoffungi number: FoF09174 (Figure 2)

Etymology: Referring to the genus of palm trees *Elaeis* Jacq.

Holotype: MFLU 20-0522.

*Saprobic* on dead leaves and rachis of *Elaeis guineensis*. Sexual morph: *Ascomata* 160–280 × 130–350 μm (*x¯* = 220 × 240 μm, n = 20), immersed in the host epidermis, beneath a clypeus, visible as slightly raised blackened areas, dark brown to black, coriaceous, solitary or aggregated into clusters, scattered, with an ostiolar canal. *Peridial wall* 13–45 μm wide, thick, comprising several layers of cells, outwardly comprising dark brown cells of *textura irregularis* and inwardly comprising hyaline cells of *textura prismatica*, 7–20 μm wide. *Paraphyses* 1.5–4.5 μm wide, septate, hyphae-like, hyaline. *Asci* 50–90 × 10–15 μm (*x¯* = 70 × 12 μm, n = 40), 8-spored, unitunicate, clavate to cylindric, short pedicellate, with J- apical ring. *Ascospores* 10–18 × 5–8 μm (*x¯* = 14 × 6 μm, n = 100), uni–biseriate into the asci, unicellular, obovoid, fusoid, hyaline to light brown when immature and brown to dark brown when mature, mostly one, rarely two-guttulate, cell wall verrucose, with a mucilaginous cap at the apex. *Germslit* 3–6 μm length (*x¯* = 5 μm, n = 50), straight, less than spore-length. Asexual morph: Not observed.

Material examined: THAILAND, Surat Thani Province, on dead leaves and rachis of *Elaeis guineensis* Jacq. (Arecaceae) on the ground, 21 July 2017, Sirinapa Konta, SRWD12 (MFLU 20-0522, holotype).

Notes: A BLAST search of *H. elaeidis* ITS sequence shows 83.87% similarity with *Gyrothrix oleae* (CPC 37069); LSU sequence shows 95.95% similarity with *Gyrothrix eucalypti* (CPC 36066); and *rpb2* sequence shows 80.95% similarity with *Lopadostoma meridionale* (LG). Only the sexual morph of *H. elaeidis* was found in nature, and we could not obtain a pure culture from fresh samples. Therefore, the morphological characteristics of *H. elaeidis* were not compared with *Ceratocladium*, *Circinotrichum*, *Gyrothrix*, and *Idriella*, as they only had asexual morphs found in nature. Hence, the morphological features of *H. elaeidis* were only compared with *Neoanthostomella*, *Virgaria*, and *Xenoanthostomella*, as they have sexual morphs.**Key to genera related to *Anthostomella*-like genera**1. Hyaline ascospores***Alloanthostomella***1. Brown ascospores22. Asci with a J- apical ring32. Asci with or without J+ apical ring53. Ascospores with or without germ slit43. Ascospores with germ slit***Xenoanthostomella***4. Ascospores with a germ slit and the length less than spore length with a mucilaginous cap at the apex***Haploanthostomella***4. Ascospores with or without germ slit, with mucilaginous sheath***Neoanthostomella***5. Asci with a J+ apical ring, ascospores with germ slit, with or without mucilaginous sheath65. Asci with J+ or J- apical ring, ascospores with or without germ slit (straight or spiral), and also with or without appendages or mucilaginous sheath***Anthostomella***6. Ascospores with germ slit less than spore length, with or without mucilaginous sheath*7*6. Ascospores with germ slit extending over full length with mucilaginous sheath***Pseudoanthostomella***7. Ellipsoid ascospores without mucilaginous sheath***Virgaria***7. Inequilaterally oblong-ellipsoidal ascospores with mucilaginous sheath***Anthostomelloides***

#### 3.1.3. ***Endocalyx*** Berk. & Broome, J. Linn. Soc., Bot. 15(1): 84 (1876) [1877]

Index Fungorum number: IF8158; Facesoffungi number: FoF09175

*Saprobic* on various plants. *Colonies* on host plant, pustules nearly flat or raised, circular, discolored, dark brown to black, at last bursting, the conidiomata developing. Sexual morph: Undetermined. Asexual morph: *Conidiomata* scattered, erect, cupulate to cylindrical; peridial hyphae enclosing the inner conidial mass, nonsporiferous, brown to yellowish brown; some species consisting of two parts of conidioma: (1) a basal cylinder covering a central column, rough-walled, carbonaceous, composed of black hyphae which are sometimes branched and are adherent to one another; (2) a slender central column, synnematous, expanding radially apically, high, enclosed by the peridial hyphae which are nonsporiferous, orange-yellow to lemon-yellow. *Peridial wall* thick, comprising dark brown, thick-walled cells of *textura angularis*. *Conidiophores* thread-like, septate, with or without short pegs bearing the conidia, meristematic at the base, colorless basally and gradually turning brown apically, 1–2 µm wide; *peridium* thick, comprising dark brown, thick-walled cells of *textura angularis*. *Conidiogenous cells* holoblastic, integrated, determinate. *Conidia* solitary, unicellular, flattened, round, oval or slightly polygonal in face view, at first pale, dark brown to fuscous black at maturity, with or without guttules, often with a longitudinal hyaline straight germ slit extending the full-length (adapted from [99,100,101]).

Type species: *Endocalyx thwaitesii* Berk. & Broome

Notes: *Endocalyx* is a coelomycetous genus in Cainiaceae with *E. cinctus* collected from Japan *E. metroxyli* sp. nov. collected from Thailand. Phylogenetic analyses of a single dataset of ITS (supporting information section) and phylogenetic analyses of a combined dataset of ITS, LSU, *rpb2*, and *tub2* regions (Figure 1) confirm the placement of *Endocalyx* within Cainiaceae. ITS analyses showed that *Endocalyx* is closely related to *Amphibambusa* and *Atrotorquata* (supporting information section), while Figure 1 shows that *Endocalyx* formed a basal clade to other cainiaceous genera (*Alishanica*, *Amphibambusa*, *Arecophila*, *Atrotorquata*, *Cainia*, *Longiappendispora*, and *Seynesia*) with high bootstrap support. Morphologically, *Endocalyx* has been revised and described only as an asexual morph of the genus [100,101], while all genera in Cainiaceae have been described in their sexual morphs, except the type genus *Cainia,* for which both asexual and sexual morphs have been described. We could not compare the morphology of *Endocalyx* to *Arecophila*, *Seynesia*, and *Amphibambusa* (sister species in Figure 1). Therefore, *Cainia* was used for morphological comparisons; *Endocalyx* differs from *Cainia* in having erect conidiomata and also the ostiole opening surrounded by yellow hyphae, ellipsoid-globose conidia, unicellular with brown to dark brown color, and a germ slit. *Cainia* has immersed conidiomata, conidiogenous cells with one to three phialides, and elongate fusiform conidia, unicellular or septate, hyaline, with pointed ends [100,101,102].

Recently, *Longiappendispora* was introduced under Cainiaceae, with seven genera in total included in the family by Mapook et al. [11]. In our study, detailed molecular analyses were done for *Endocalyx* and its placement in Cainiaceae (Xyalriales) was confirmed. Previously, *Endocalyx* was classified in Apiosporaceae (Xylariales, Sordariomycetes) based on morphological evidence. As the first detailed molecular data of *Endocalyx cinctus* have been made available from a Japan laboratory [32], their current placement is supported (Figure 1). However, there are no recent publications referring to the molecular data of this genus yet. Thus, in this study, we present the placement of *Endocalyx* based on multigene phylogenetic analyses with recent sequence data from the Japan collection as well as the Thailand collection. In addition, we accept eight genera in Cainiaceae (*Alishanica*, *Amphibambusa*, *Arecophila*, *Atrotorquata*, *Cainia*, *Endocalyx*, *Longiappendispora*, and *Seynesia*), and seven species by including our new species in the genus *Endocalyx* (Table 2). In addition, we provide a key for the members of Cainiaceae.

#### 3.1.4. ***Endocalyx metroxyli*** Konta & K.D. Hyde. sp. nov.

**Figure 3:**
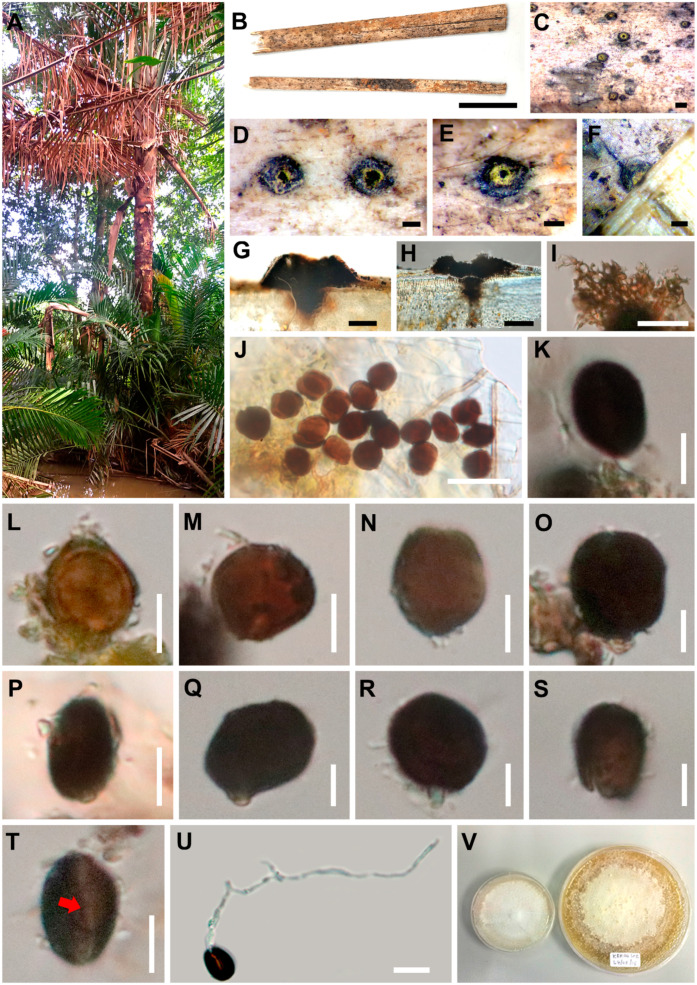
*Endocalyx metroxyli* (MFLU 15-1454, holotype). (**A**) Forest in Krabi Province. (**B**) Palm samples. (**C**–**E**) Appearance of conidiomata on host. (**F**) Vertical cut of a conidioma. (**G**–**H**) Vertical section of a conidioma. (**I**) Section of peridium. (**J**) Group of conidia. (**K**) Conidiophores reduced to conidiogenous cell with conidium. (**L**–**S**) Conidia (**P**–**R**, Conidia with conidiogenous cells). (**T**) Germ slit (red arrow). (**U**) Germinated conidia. (**V**) Colonies on MEA media. Scale bars: B = 2 cm, C = 500 μm, D–H = 200 μm, I, J = 20 μm, L–T = 5 μm, U = 10 μm.

Index Fungorum number: IF558116, Facesoffungi number: FoF09176 (Figure 3)

Etymology: Refers to the name of the host genus, *Metroxylon*.

Holotype: MFLU 15-1454.

*Saprobic* on dead petiole of *Metroxylon sagu*. *Colonies* on host plant, pustules. Sexual morph: Undetermined. Asexual morph: *Conidiomata* 340–660 μm wide, in vertical section 495–820 × 325–485 µm, acervulus, solitary, semi-immersed to immersed in the host epidermis, beneath a clypeus, visible as slightly raised and blackened, black, carbonaceous, fragile, with an ostiolar canal. *Ostiolar* opening surrounded by a yellow margin. *Peridial wall* 34–80 μm wide, thick, comprising dark brown cells of *textura angularis*. *Conidiomata* not observed with a basal cylinder covering a central column or a slender central column in our collection. *Conidiophores* reduced to conidiogenous cell, hyaline to pale-brown, unbranched, smooth. *Conidia* 13–16 × 7–10 µm (*x¯* = 13 × 10 µm, n = 30), unicellular, ellipsoid-globose, brown to dark brown, with short pegs bearing conidia, with germ slit, smooth-walled.

Culture characteristics: Colonies on MEA, at first white, raised, effuse, velvety to hairy, circular, smooth at the margin, white from above, pale-brown from below.

Material examined: Thailand, Krabi Province, on dead petiole of *Metroxylon sagu* Rottb. on the ground (Arecaceae), 8 December 2014, Sirinapa Konta KBR04h2 (MFLU 15-1454, holotype); ex-type living culture, MFLUCC 15-0723A; *ibid*. MFLUCC 15-0723B, MFLUCC 15-0723C.

Additional sequence data: SSU: MT929310, MT929311, *tef1-α*: MT928152, MT928153.

Notes: *Endocalyx metroxyli* is phylogenetically well supported and is placed in Cainiaceae (Figure 1). *Endocalyx metroxyli* is closely related to *E. cinctus* with high bootstrap support but is distinct in morphological characteristics. A BLAST search of *E. metroxyli* ITS sequence shows 83.10% similarity with *Requienella seminuda* (CBS 140502) (CPC 37069), LSU sequence shows 96.14% similarity with *Entosordaria quercina* (RQ), *tub2* sequence shows 88.94% similarity with *Daldinia dennisii var. dennisii*, SSU sequence shows 97.92% similarity with *Xenoanthostomella chromolaenae* (MFLUCC 17-1484), and *tef1-α* sequence shows 89.39% similarity with *Barrmaelia macrospor* (BM).

*Endocalyx metroxyli* is morphologically similar to *E. melanoxanthus*. However, *Endocalyx metroxyli* does not have erect conidiomata developing from the pustules, as was mentioned by Petch [100], Okada and Tubaki [101], and Vitoria et al. [102,131]. In this study, we found only a black raised pustule structure with ostiole surrounded by a yellow hyphae ring, and hyaline conidiophore, unicellular, dark brown conidia with a longitudinal germ slit. *Endocalyx melanoxanthus* was collected and described from palm hosts (Arecaceae), and a few collections were collected from other host plants (Table 2). According to Species Fungorum [134], *E. melanoxanthus var. Grossus* (G. Okada & Tubaki) and *E. melanoxanthus var. melanoxanthus* (Berk. & Broome) are considered as *E. melanoxanthus*, even though they have several different characteristics.

*Endocalyx metroxyli* is morphologically similar to *E. melanoxanthus var. melanoxanthus*, in having black raised pustules surrounded by yellow hyphae and smooth-walled conidia with no significant size differences [100,101,102]. However, our new taxon lacks cupulate or cylindrical conidiomata [101,102]. On the other hand, *E. metroxyli* differs from *E. melanoxanthus var. grossus* by lacking the production of ornamented conidia [100,101].
**Keys to genera of Cainiaceae****1. Asexual morph**1.1 Coelomycetous; 1–3 phialides conidiogenous cells, and elongate fusiform conidia with unicellular or septate, with pointed ends***Cainia***1.1 Coelomycetous; conidiomata with ostiolar opening surrounded by yellow, with unicellular conidia, ellipsoid-globose, pale to dark brown to black, with a straight germ slit extending the full-length***Endocalyx*****2. Sexual morph**2.1 Cylindrical-clavate asci, ascospores with 1-septate(2.2)2.1 Cylindrical, or cylindrical to elongate cylindrical asci, ascospores with 1-septate(2.3)2.2 Ellipsoidal ascospores, with brown, and sheath***Cainia***2.2 Ellipsoidal to fusiform ascospores, with brown, and sheath***Atrotorquata***2.3 Ellipsoid to broadly fusiform ascospores, longitudinal striations, bristle-like polar appendages from both ends, without a gelatinous sheath***Longiappendispora***2.3 Fusiform to broad-fusiform ascospores with pointed at both ends, striation wall, and sheath***Amphibambusa***2.3 Ellipsoidal or oblong ascospores(2.4)2.4 Oblong ascospores with cap-like appendage, germ slits***Seynesia***2.4 Ellipsoidal ascospores(2.5)2.5 Ascospores with striation wall, brown, and sheath***Alishanica***2.5 Ascospores with striate or verrucose wall, and subhyaline to brown***Arecophila***

## 4. Discussion

Based on phylogeny and morphological characteristics, the new monotypic genus *Haploanthostomella* (type species: *Haploanthostomella elaeidis*) and the new species *Endocalyx metroxyli* have been established. The former new species was isolated from a dead rachis of *Elaeis guineensis*, and the latter from a dead petiole of *Metroxylon sagu* (Arecaceae) in Thailand. Phylogenetic analyses of combined datasets together with morphological characteristics revealed that *Haploanthostomella* belongs to Xylariales *incertae sedis*, while *Endocalyx* belongs to the Cainiaceae (Xylariales).

Based on morphological features, *Endocalyx* was assigned to Apiosporaceae (Amphisphaeriales, Sordariomycetes), together with four other genera, viz. *Appendicospora*, *Arthrinium*, *Dictyoarthrinium*, and *Nigrospora* [3,8]. Later, *Dictyoarthrinium* was transferred to Didymosphaeriaceae (Pleosporales, Dothideomycetes) [135]. According to our phylogenetic analyses (Figure 1), *Arthrinium* and *Nigrospora* should be accepted under the Apiosporaceae, while *Appendicospora* did not clade to this family (supporting information section), and *Endocalyx* fits well within the Cainiaceae.

Interestingly, four out of seven species in the genus *Endocalyx* (*E. melanoxanthus*, *E. cinctus*, *E. indumentum*, and *E. thwaitesii*) were collected from palm hosts (Table 2). *Endocalyx metroxyli* is similar to other species by having dark brown conidia with a full-length germ slit, it but differs from other species by not having conidiomata produced from the pustulate and no thread-like structure of conidiophores. Morphological characteristics of species in the genus are mostly flat or raised pustules, capsule or slender conidiomata with or without branches at the apex, and brown to dark brown conidia with smooth walls (*E. amarkantakensis*, *E. collantesis*, *E. indumentum*, *E. melanoxanthus*, *E. melanoxanthus var. melanoxanthus*), while some species are verrucose-walled (*E. cinctus*, *E. indumentum*, *E. melanoxanthus var. grossus*, *E. thwaitesii*). We referred to previous publications for morphological comparisons to the taxa in this study, as we did not observe all holotype specimens [100,101,102].

According to the literature, there are also strains derived from another two species and two varieties. Excluding *E. cinctus*, no sequence data are available for generic types of *Endocalyx* and other species, and their morphology and host substrates are closely related to our novel taxon. *Endocalyx* species have been reported in several countries, especially in tropical and subtropical regions. Furthermore, palm trees (Arecaceae) have most commonly been reported as the host, while several species have been presented from other hosts (Table 2).

The phylogenetic placement of many groups within the Xylariales remains unclear (e.g., *Anthostomelloides*, *Calceomyces*, *Circinotrichum*, *Fasciatispora* (only *F. petrakii*), *Gyrothyrix*, *Melanographium*, *Neoanthostomella*, *Pseudoanthostomella*, and *Xenoanthostomella*, Figure 1). Thus, it is necessary to collect and analyze more fungal specimens from Xylariales using multigene phylogeny (with protein coding genes) and morphology to resolve their taxonomical placement and delimitation.

## Figures and Tables

**Table 1:** Names, strain numbers and corresponding GenBank accession numbers of the taxa used in phylogenetic analyses, the ex-type strains are in bold.

Order	Family	Species	Strain No.	GenBank Accession No.				References
				ITS	LSU	*rpb2*	*tub2*	
Amphisphaeriales	Apiosporaceae	***Arthrinium balearicum***	AP24118	MK014869	MK014836	-	MK017946	[21]
Amphisphaeriales	Apiosporaceae	*Arthrinium caricicola*	CBS 145127	MK014871	MK014838	-	MK017948	[21]
Amphisphaeriales	Apiosporaceae	***Arthrinium hydei***	CBS 114990	KF144890	KF144936	-	KF144982	[22]
Amphisphaeriales	Apiosporaceae	***Arthrinium phragmitis***	CBS 135458	KF144909	KF144956	-	KF145001	[22]
Amphisphaeriales	Apiosporaceae	***Arthrinium pseudospegazzinii***	CBS 102052	KF144911	KF144958	-	KF145002	[22]
Amphisphaeriales	Apiosporaceae	***Nigrospora aurantiaca***	CGMCC 3.18130	NR_153477	NG_069394	-	KY019465	[23]
Amphisphaeriales	Apiosporaceae	***Nigrospora brasiliensis***	CMM 1214	KY569629	-	-	MK720816	[24]
Amphisphaeriales	Apiosporaceae	***Nigrospora zimmermanii***	CBS 290.62	KY385309	-	KY806276	KY385317	[23]
Amphisphaeriales	Beltraniaceae	***Beltrania rhombica***	CBS 123.58 = IMI 072432	MH553990	MH554209	MH554899	MH704631	[25]
Amphisphaeriales	Beltraniaceae	***Beltraniella endiandrae***	CBS 137976	KJ869128	KJ869185	-	-	[26]
Amphisphaeriales	Beltraniaceae	***Beltraniopsis neolitseae***	CBS 137974	KJ869126	KJ869183	-	-	[26]
Amphisphaeriales	Beltraniaceae	***Arecophila bambusae***	HKUCC 4794	-	AF452038	-	-	[27]
Xylariales	Cainiaceae	***Alishanica miscanthii***	FU31025	MK503821	MK503827	-	-	[3]
Xylariales	Cainiaceae	***Amphibambusa bambusicola***	MFLUCC 11-0617	KP744433	KP744474	-	-	[28]
Xylariales	Cainiaceae	***Atrotorquata lineata***	HKUCC 3263	AF009807	-	-	-	Unpublished
Xylariales	Cainiaceae	***Cainia anthoxanthis***	MFLUCC 15-0539	KR092787	KR092777	-	-	[5]
Xylariales	Cainiaceae	***Cainia desmazieri***	CAI	KT949896	KT949896	-	-	[29]
Xylariales	Cainiaceae	***Cainia globosa***	MFLUCC 13-0663	KX822127	KX822123	-	-	[30]
Xylariales	Cainiaceae	***Cainia graminis***	CBS 136.62	KR092793	AF431949	-	-	[5,31]
Xylariales	Cainiaceae	***Longiappendispora chromolaenae***	MFLUCC 17-1485	MT214370	MT214464	-	-	[11]
Xylariales	Cainiaceae	***Endocalyx cinctus***	JCM 7946	LC228648	LC228704	-	-	[32]
Xylariales	Cainiaceae	***Endocalyx metroxyli***	MFLUCC 15-0723A	MT929162	MT929313	-	-	This study
Xylariales	Cainiaceae	***Endocalyx metroxyli***	MFLUCC 15-0723B	MT929163	MT929314	-	MT928155	This study
Xylariales	Cainiaceae	***Endocalyx metroxyli***	MFLUCC 15-0723C	-	MT929315	-	-	This study
Xylariales	Cainiaceae	***Seynesia erumpens***	SMH 1291	-	AF279410	-	-	[33]
Xylariales	Clypeosphaeriaceae	*Clypeosphaeria mamillana*	CBS 140735	KT949897	KT949897	MF489001	MH704637	[29,34]
Xylariales	Coniocessiaceae	***Coniocessia anandra***	Co108	GU553338	GU553349	-	-	[35]
Xylariales	Coniocessiaceae	***Coniocessia cruciformis***	Co116	GU553336	GU553347	-	-	[35]
Xylariales	Coniocessiaceae	***Coniocessia maxima***	Co117	GU553332	GU553344	-	-	[35]
Xylariales	Coniocessiaceae	***Coniocessia minima***	Co111	GU553334	GU553345	-	-	[35]
Xylariales	Coniocessiaceae	***Coniocessia nodulisporioides***	CBS 281.77T	-	AJ875224	-	-	[36]
Xylariales	Coniocessiaceae	***Paraxylaria rosacearum***	TASM 6132	MG828941	MG829050	-	-	[37]
Xylariales	Diatrypaceae	***Allocryptovalsa polyspora***	MFLUCC 17-0364	MF959500	MF959503	-	MG334556	[38]
Xylariales	Diatrypaceae	***Allodiatrype arengae***	MFLUCC 15-0713	MN308411	MN308402	MN542886	MN340297	[39]
Xylariales	Diatrypaceae	***Cryptovalsa rabenhorstii***	CreI = CBS 125574	KC774567	KC774567	-	-	[40]
Xylariales	Diatrypaceae	*Diatrype disciformis*	CBS 197.49	-	DQ470964	DQ470915	-	[41]
Xylariales	Diatrypaceae	***Diatrypella verruciformis***	UCROK1467	JX144793	-	-	JX174093	[42]
Xylariales	Diatrypaceae	***Eutypa lata***	CBS 208.87	DQ006927	MH873755	-	DQ006969	[43,44]
Xylariales	Diatrypaceae	***Eutypella caricae***	EL5C	AJ302460	-	-	-	[45]
Xylariales	Diatrypaceae	***Halodiatrype salinicola***	MFLUCC 15-1277	KX573915	-	-	KX573932	[46]
Xylariales	Diatrypaceae	***Monosporascus cannonballus***	CMM3646	JX971617	-	-	-	Unpublished
Xylariales	Diatrypaceae	***Neoeutypella baoshanensis***	EL51C, CBS 274.87	AJ302460	-	-	-	[45]
Xylariales	Diatrypaceae	***Pedumispora rhizophorae***	BCC44877	KJ888853	KJ888850	-	-	[47]
Xylariales	Diatrypaceae	***Peroneutypa longiasca***	MFLUCC 17-0371	MF959502	MF959505	-	MG334558	[38]
Xylariales	Fasciatisporaceae	***Fasciatispora arengae***	MFLUCC 15-0326a	MK120275	MK120300	MK890794	MK890793	[48]
Xylariales	Fasciatisporaceae	***Fasciatispora calami***	MFLUCC 15-0294	-	MF459055	-	MF459056	[49]
Xylariales	Fasciatisporaceae	***Fasciatispora cocoes***	MFLUCC 18-1445	MN482680	MN482675	MN481517	MN505154	[13]
Xylariales	Fasciatisporaceae	***Fasciatispora nypae***	MFLUCC 11-0382	-	KP744484	-	-	[28]
Xylariales	Fasciatisporaceae	***Fasciatispora petrakii***		-	AY083828	-	-	Unpublished
Xylariales	Graphostromataceae	*Biscogniauxia nummularia*	MUCL 51395	KY610382	KY610427	KY624236	KX271241	[50]
Xylariales	Graphostromataceae	*Camillea obularia*	ATCC 28093	KY610384	KY610429	KY624238	KX271243	[50]
Xylariales	Graphostromataceae	*Graphostroma platystomum*	CBS 270.87	JX658535	DQ836906	KY624296	HG934108	[50,51,52,53]
Xylariales	Graphostromataceae	*Obolarina dryophila*	MUCL 49882	GQ428316	GQ428316	KY624284	GQ428322	[50,54]
Xylariales	Hansfordiaceae	***Hansfordia pulvinate***	CBS 194.56	MK442585	MH869122	KU684307	-	[24]
Xylariales	Hansfordiaceae	*Hansfordia pulvinate*	CBS 144422	MK442587	MK442527	-	-	[24]
Xylariales	Hypoxylaceae	*Annulohypoxylon truncatum*	CBS 140778	KY610419	KY610419	KY624277	KX376352	[50,55]
Xylariales	Hypoxylaceae	***Anthocanalis sparti***	MFLUCC 14-0010	KP297394	KP340536	KP340522	KP406605	[54]
Xylariales	Hypoxylaceae	***Anthostoma decipiens***	CD = CBS 133221	KC774565	KC774565	-	-	[40]
Xylariales	Hypoxylaceae	*Daldinia concentrica*	CBS 113277	AY616683	KY610434	KY624243	KC977274	[50,56,57]
Xylariales	Hypoxylaceae	*Durotheca depressa*	BCC28073	-	-	-	GQ160492	[58]
Xylariales	Hypoxylaceae	*Entonaema liquescens*	ATCC 46302	KY610389	KY610443	KY624253	KX271248	[50]
Xylariales	Hypoxylaceae	***Hypomontagnella monticulosa***	MUCL 54604	KY610404	KY610487	KY624305	KX271273	[50]
Xylariales	Hypoxylaceae	*Hypoxylon fragiforme*	MUCL 51264	KC477229	KM186295	KM186296	KX271282	[50,59,60]
Xylariales	Hypoxylaceae	*Jackrogersella multiformis*	CBS 119016	KC477234	KY610473	KY624290	KX271262	[50,55,57]
Xylariales	Hypoxylaceae	*Pyrenomyxa morganii*	CBS 116990T	AM749920	-	-	-	[61]
Xylariales	Hypoxylaceae	*Pyrenomyxa picea*	ILLS 58257	-	EF562506	-	-	[62]
Xylariales	Hypoxylaceae	*Pyrenopolyporus hunteri*	MUCL 52673	KY610421	KY610472	KY624309	KU159530	[50,55]
Xylariales	Hypoxylaceae	*Rhopalostroma indicum*	CBS 113035	MH862909	MH874483	-	-	[44]
Xylariales	Hypoxylaceae	*Thamnomyces dendroidea*	CBS 123578	FN428831	KY610467	KY624232	KY624313	[50,63]
Xylariales	Hypoxylaceae	*Thuemenella cubispora*	CBS 119807	JX658531	EF562508	-	-	[62]
Xylariales	Hypoxylaceae	*Phylacia sagrana*	CBS 119992	AM749919	-	-	-	[61]
Xylariales	Hypoxylaceae	***Pyrenopolyporus symphyon***	TBRC:8873	MH938529	MH938538	MK165428	MK165419	[64]
Xylariales	Induratiaceae	*Emarcea castanopsidicola*	CBS 117105	MK762710	MK762717	MK791285	MK776962	[64]
Xylariales	Induratiaceae	*Emarcea eucalyptigena*	CBS 139908	MK762711	MK762718	MK791286	MK776963	[64]
Xylariales	Induratiaceae	***Induratia fengyangensis***	CGMCC 2862	HM034856	HM034859	HM034849	HM034843	[65]
Xylariales	Induratiaceae	***Induratia thailandica***	MFLUCC 17-2669	MK762707	MK762714	MK791283	MK776960	[64]
Xylariales	Lopadostomataceae	*Creosphaeria sassafras*	STMA 14087	KY610411	KY610468	KY624265	KX271258	[50]
Xylariales	Lopadostomataceae	*Lopadostoma turgidum*	CBS 133207	KC774618	KC774618	KC774563	MF489024	[29,40]
Xylariales	Microdochiaceae	***Idriella lunata***	MUCL 4103	KC775734	KC775709	-	-	[66]
Xylariales	Microdochiaceae	***Idriella lunata***	CBS 204.56	KP859044	KP858981	-	-	[67]
Xylariales	Microdochiaceae	***Microdochium phragmitis***	CBS 423.78	KP859012	KP858948	KP859121	KP859076	[67]
Xylariales	Polystigmataceae	***Polystigma fulvum***	MFLU 18-0261	MK429738	MK429727	-	-	[68]
Xylariales	Polystigmataceae	***Polystigma rubrum***	MFLU 15-3091	KY594023	MF981079	-	-	[68]
Xylariales	Requienellaceae	***Acrocordiella occulta***	RS9	KT949893	KT949893	-	-	[29]
Xylariales	Requienellaceae	***Acrocordiella omanensis***	SQUCC 15091	MG584568	MG584570	-	-	[69]
Xylariales	Requienellaceae	***Requienella fraxini***	RS2	KT949909	KT949909	-	-	[29]
Xylariales	Requienellaceae	***Requienella seminuda***	RS12 = CBS 140502	KT949912	KT949912	MK523300	-	[29,64]
Xylariales	Xylariaceae	***Abieticola koreana***	EML-F0010-1	JN977612	JQ014618	KP792128	KP792126	[70]
Xylariales	Xylariaceae	*Amphirosellinia nigrospora*	HAST 91092308	GU322457	-	GQ848340	GQ495951	[71]
Xylariales	Xylariaceae	*Anthostomella formosa*	MFLUCC 14-0170	KP297403	KP340544	KP340531	KP406614	[59]
Xylariales	Xylariaceae	*Anthostomella helicofissa*	MFLUCC 14-0173	KP297406	KP340547	KP340534	KP406617	[59]
Xylariales	Xylariaceae	*Anthostomella obesa*	MFLUCC 14-0171	KP297405	KP340546	KP340533	KP406616	[59]
Xylariales	Xylariaceae	*Anthostomella pseudobambusicola*	MFLUCC 15-0192	KU940153	KU863141	-	-	[72]
Xylariales	Xylariaceae	***Anthostomelloides brabeji***	CBS 110128	EU552098	EU552098	-	-	[73]
Xylariales	Xylariaceae	***Anthostomelloides forlicesenica***	MFLUCC 14-0558	KP297397	KP340539	-	KP406608	[66]
Xylariales	Xylariaceae	***Anthostomelloides krabiensis***	MFLUCC 15-0678	KX305927	KX305928	KX305929	-	[30]
Xylariales	Xylariaceae	***Anthostomelloides leucospermi***	CBS:110126	EU552100	-	-	-	[73]
Xylariales	Xylariaceae	***Anthostomelloides proteae***	CBS 110127	EU552101	-	-	-	[73]
Xylariales	Xylariaceae	***Astrocystis mirabilis***	94070803 HAST	GU322448	-	GQ844835	GQ495941	[71]
Xylariales	Xylariaceae	***Brunneiperidium gracilentum***	MFLUCC 14-0011 Ex-type	KP297400	KP340542	KP340528	KP406611	[66]
Xylariales	Xylariaceae	*Collodiscula japonica*	CBS 124266	JF440974	JF440974	KY624273	KY624316	[50,74]
Xylariales	Xylariaceae	***Coniolariella gamsii***	Co27IRAN 842C, CBS114379 (T)	GU553325	GU553329	-	-	[35]
Xylariales	Xylariaceae	*Entalbostroma erumpens*	ICMP 21152	KX258206	-	KX258204	KX258205	[75]
Xylariales	Xylariaceae	*Entoleuca mammata*	J.D.R. 100	GU300072	-	GQ844782	GQ470230	[71]
Xylariales	Xylariaceae	*Euepixylon sphaeriostomum*	J.D.R. 261	GU292821	-	GQ844774	GQ470224	[71]
Xylariales	Xylariaceae	*Halorosellinia oceanica*	SGLAf82	EU715635	-	-	-	[76]
Xylariales	Xylariaceae	*Hypocopra rostrata*	NRRL 66178	KM067909	-	-	-	[77]
Xylariales	Xylariaceae	*Hypocreodendron sanguineum*	J.D.R. 169	GU322433	-	GQ844819	GQ487710	[71]
Xylariales	Xylariaceae	***Kretzschmaria clavus***	YMJ 114	EF026126	-	GQ844789	EF025611	[71,78]
Xylariales	Xylariaceae	***Linosporopsis ischnotheca***	LIF1 = CBS 145761	MN818952	MN818952	MN820708	MN820715	[79]
Xylariales	Xylariaceae	***Lunatiannulus irregularis***	MFLUCC 14-0014	KP297398	KP340540	KP340526	KP406609	[57]
Xylariales	Xylariaceae	*Nemania serpens*	CBS 679.86	KU683765	-	KU684284	KU684188	[80]
Xylariales	Xylariaceae	***Neoxylaria arengae***	MFLUCC 15-0292	MT496747	-	MT502418	-	[81]
Xylariales	Xylariaceae	***Podosordaria mexicana***	WSP 176	GU324762	-	GQ853039	GQ844840	[71]
Xylariales	Xylariaceae	***Poronia punctata***	CBS 656.78	KT281904	KY610496	KY624278	KX271281	[5,50]
Xylariales	Xylariaceae	*Rosellinia aquila*	MUCL 51703	KY610392	KY610460	KY624285	KX271253	[50]
Xylariales	Xylariaceae	*Rostrohypoxylon terebratum*	CBS 119137	DQ631943	DQ840069	DQ631954	DQ840097	[82,83]
Xylariales	Xylariaceae	*Ruwenzoria pseudoannulata*	MUCL 51394	KY610406	KY610494	KY624286	KX271278	[50]
Xylariales	Xylariaceae	*Sarcoxylon compunctum*	CBS 359.61	KT281903	KY610462	KY624230	KX271255	[5,50]
Xylariales	Xylariaceae	*Stilbohypoxylon elaeicola*	Y.M.J. 173	EF026148	-	GQ844826	EF025616	[71]
Xylariales	Xylariaceae	***Stilbohypoxylon elaeidis***	MFLUCC 15-0295a	MT496745	MT496755	MT502416	MT502420	[81]
Xylariales	Xylariaceae	*Stilbohypoxylon quisquiliarum*	Y.M.J. 172	EF026119	-	GQ853020	EF025605	[71]
Xylariales	Xylariaceae	***Vamsapriya bambusicola***	MFLUCC 11-0477	KM462835	KM462836	KM462834	KM462833	[84]
Xylariales	Xylariaceae	***Vamsapriya breviconidiophora***	MFLUCC 14-0436	MF621584	MF621588	-	-	[39]
Xylariales	Xylariaceae	***Vamsapriya indica***	MFLUCC 12-0544	KM462839	KM462840	KM462841	KM462838	[84]
Xylariales	Xylariaceae	***Vamsapriya khunkonensis***	MFLUCC 11-0475	KM462830	KM462831	KM462829	KM462828	[84]
Xylariales	Xylariaceae	***Vamsapriya yunnana***	KUMCC 18-0008	MG833874	MG833873	MG833875	-	[85]
Xylariales	Xylariaceae	***Virgaria boninensis***	JCM 18624	AB740956	AB740960	-	-	[86]
Xylariales	Xylariaceae	***Virgaria nigra***	CBS 128006	MH864744	MH876180	-	-	[44]
Xylariales	Xylariaceae	*Xylaria hypoxylon*	CBS 122620	KY610407	KY610495	KY624231	KX271279	[50,87]
Sordariomycetes genera *incertae sedis*	Xylariales genera *incertae sedis*	***Melanographium phoenicis***	MFLUCC 18-1481	MN482677	MN482678	-	-	[13]
Sordariomycetes genera *incertae sedis*	Xylariales genera *incertae sedis*	*Ceratocladium microspermum*	CBS126092	MH864077	MH875534	-	-	[44]
Xylariales	Xylariales genera *incertae sedis*	***Ascotricha chartarum***	CBS 234.97	KF893284	-	-	KF893271	[88]
Xylariales	Xylariales genera *incertae sedis*	***Ascotricha longipila***	OUCMBI110118 (T)	KC503896	-	-	KF893265	[88]
Xylariales	Xylariales genera *incertae sedis*	***Ascotricha lusitanica***	CBS 462.70 (IT)	KF893289	-	-	KF893275	[88]
Xylariales	Xylariales genera *incertae sedis*	***Ascotricha parvispora***	OUCMBI110001 (T)	JX014298	-	-	KF893267	[88]
Xylariales	Xylariales genera *incertae sedis*	***Ascotricha sinuosa***	OUCMBI101190 (T)	JX014299	-	-	KF893266	[88]
Xylariales	Xylariales genera *incertae sedis*	***Alloanthostomella rubicola***	MFLUCC 14-0175	KP297407	KP340548	KP340535	KP406618	[89]
Xylariales	Xylariales genera *incertae sedis*	*Circinotrichum cycadis*	CPC 17285	KJ869121	KJ869178	-	-	[26]
Xylariales	Xylariales genera *incertae sedis*	*Circinotrichum maculiforme*	CPC 24566	KR611874	KR611895	-	-	[90]
Xylariales	Xylariales genera *incertae sedis*	*Circinotrichum papakurae*	CBS 101373	KR611876	KR611897	-	-	[90]
Xylariales	Xylariales genera *incertae sedis*	*Circinotrichum sinense*		KY994106	KY994107	-	-	[91]
Xylariales	Xylariales genera *incertae sedis*	***Gyrothrix eucalypti***	CPC 36066	MN562109	MN567617	-	-	[92]
Xylariales	Xylariales genera *incertae sedis*	***Gyrothrix inops***	BE108	KC775746	KC775721	-	-	[66]
Xylariales	Xylariales genera *incertae sedis*	***Gyrothrix oleae***	CPC 37069	MN562136	MN567643	-	-	[92]
Xylariales	Xylariales genera *incertae sedis*	***Gyrothrix ramosa***	MUCL54061	KC775747	KC775722	-	-	[66]
Xylariales	Xylariales genera *incertae sedis*	***Haploanthostomella elaeidis***	MFLU 20-0522	MT929161	MT929312	MT928154	-	This study
Xylariales	Xylariales genera *incertae sedis*	*Neoanthostomella pseudostromatica*	MFLUCC 11-0610	KU940158	KU863146	-	-	[72]
Xylariales	Xylariales genera *incertae sedis*	*Neoanthostomella viticola*	MFLUCC 16-0243	KX505957	KX505958	KX789496	KX789495	[89]
Xylariales	Xylariales genera *incertae sedis*	***Pseudoanthostomella conorum***	CBS 119333	EU552099	-	-	-	[73]
Xylariales	Xylariales genera *incertae sedis*	***Pseudoanthostomella delitescens***	MFLUCC 16-0477	KX533451	KX533452	KX789491	KX789490	[89]
Xylariales	Xylariales genera *incertae sedis*	***Pseudoanthostomella pini-nigrae***	MFLUCC 16-0478	KX533453	KX533454	KX789492	-	[89]
Xylariales	Xylariales genera *incertae sedis*	***Pseudoanthostomella sepelibilis***		AY908989	AY875645	-	-	Unpublished
Xylariales	Xylariales genera *incertae sedis*	***Xenoanthostomella chromolaenae***	MFLUCC 17-1484	MN638863	MN638848	-	-	[3]
Xylariales	Zygosporiaceae	***Zygosporium oscheoides***	MFLUCC 14-0402	MF621585	MF621589	-	-	[93]
Xylariales	Zygosporiaceae	***Zygosporium minus***	HKAS99625	MF621586	MF621590	-	-	[93]

**Table 2:** Host and locality information of *Endocalyx* reported worldwide based on the records of Species Fungorum 2021.

No.	Species	Host		Country	Reference
		Eudicots	Monocots		
1	*Endocalyx amarkantakensis*	*Shorea robusta* (Dipterocarpaceae)		India (Holotype)	[103]
2	*E. cinctus* *		*Livistona chinensis var. boninensis* (Arecaceae; solitary palm)	Japan	[104]
			*Oncosperma fasciculatum* (Arecaceae; clustering, rarely solitary palm)	Japan	[101]
			*Oncosperma* sp. (Arecaceae; clustering, rarely solitary palm)	Sri Lanka (Holotype)	[100]
			*Phoenix canariensis* (Arecaceae; solitary palm)	Japan	[101]
			*Phoenix hanceana* (Arecaceae; solitary palm)	Hong Kong	[105]
			*Trachycarpus fortunei* (Arecaceae; solitary palm)	Japan	[101]
3	*E. collantesis*		Smilax sp. (Smilacaceae)	Cuba (Holotype)	[106]
4	*E. indicus*	twigs of woody		India (Holotype)	[107]
5	*E. indumentum*		*Livistona chinensis var. boninensis* (Arecaceae; solitary palm)	Japan (Holotype)	[101,104]
			*Phoenix canariensis* (Arecaceae; solitary palm)	Japan	[104]
6	*E. melanoxanthus*		*Acrocomia mexicana* (Arecaceae)	Mexico	[108]
			*Archontophoenix alexandrae* (Arecaceae; solitary palm)	Australia	[109]
				Hong Kong	[105,109]
				Malaysia	[109]
				Singapore	[109]
			Arecaceae	Mexico	[108]
			*Arenga engleri* (Arecaceae; clustering palm)	Hong Kong	[105]
				Japan	[104]
			*Dypsis lutescens* (=*Chrysalidocarpus lutescens*) (Arecaceae; clustering palm)	Japan	[104]
			*Caryota urens* (Arecaceae; solitary palm)	Sri Lanka (Holotype)	[100]
			*Cocos nucifera* (Arecaceae; solitary palm)	Australia	[109]
				Ghana	[110]
				Hawaii	[111,112]
				Japan	[104]
				Malaysia	[109,113]
				Papua New Guinea	[114]
				Seychelles	[109]
				Singapore	[109]
		*Coffea arabica* (Rubiaceae)		Venezuela	[115]
			*Dracaena fragrans* (Asparagaceae)	Cuba	[116]
				Venezuela	[115]
			*Elaeis guineensis* (Arecaceae; solitary palm)	Ghana	[110]
				Myanmar	[117]
				Sierra Leone	[113]
			*Elaeis* sp. (Arecaceae; solitary palm)	Japan	[104]
			*Licuala longicalycata* (Arecaceae; solitary palm)	Thailand	[118]
			*Livistona chinensis* (Arecaceae; solitary palm)	Hong Kong	[105]
			*Livistona chinensis var. boninensis* (Arecaceae; solitary palm)	Japan	[104]
			*Livistona rotundifolia* (Arecaceae; solitary palm)	Taiwan	[119]
			*Livistona speciosa* (Arecaceae; solitary palm)	Myanmar	[117]
			*Nannorrhops ritchieana* (Arecaceae; clustering palm)	Pakistan	[120]
			*Phoenix canariensis* (Arecaceae; solitary palm)	Japan	[104]
			*Phoenix hanceana* (Arecaceae; solitary palm)	Hong Kong	[105,121]
			*Phoenix reclinata* (Arecaceae; solitary palm)	Ghana	[110]
			*Phoenix roebelenii* (Arecaceae; solitary palm)	Japan	[104]
			*Phoenix roebelenii-senegalensis* (Arecaceae; solitary palm)	Japan	[104]
			*Ravenala madagascariensis* (Strelitziaceae)	Japan	[104]
				Taiwan	[119]
			*Ripogonum scandens* (Ripogonaceae)	New Zealand	[122]
			*Roystonea borinquena* (Arecaceae; solitary palm)	USA (Florida)	[123]
			*Roystonea* regia (Arecaceae; solitary palm)	Cuba	[124,125,126,127]
			*Sabal palmetto* (Arecaceae; solitary palm)	USA (Florida)	[128]
			*Serenoa serrulata* (Arecaceae; clustering and solitary palm)	USA (Florida)	[129]
			*Smilax* sp. (Smilacaceae)	USA (Florida)	[128]
			*Trachycarpus fortunei* (Arecaceae; solitary palm)	China	[109]
			unknown, palm	Australia	[109]
				China	[109]
				Hong Kong	[109]
				Malaysia	[109]
				Seychelles	[109]
				Singapore	[109]
			*Wodyetia bifurcata* (Arecaceae; solitary palm)	Florida	[123]
	*E. melanoxanthus* (=*E. melanoxanthus var. grossus*)		*Trachycarpus fortunei* (Arecaceae; solitary palm)	Japan	[101]
	*E. melanoxanthus* (=*E. melanoxanthus var. melanoxanthus*)		*Acrocomia intumescens* (Arecaceae; solitary palm)	Brazil	[102]
			*Butia yatay* (Arecaceae; solitary palm)	Argentina	[130]
			*Cocos nucifera* (Arecaceae; solitary palm)	Ghana	[101]
			*Euterpe edulis* (Arecaceae; solitary, or rarely clustering palm (growing in dense tufts or clumps) and then with few stems)	Argentina	[130]
				Brazil	[102]
			*Euterpe oleracea* (Arecaceae; clustering palm)	Brazil	[102]
			*Livistona chinensis var. boninensis* (Arecaceae; solitary palm)	Japan	[101]
			*Livistona chinensis var. subglobosa* (Arecaceae; solitary palm)	Japan	[101]
			*Phoenix canariensis* (Arecaceae; solitary palm)	Japan	[101]
			*Phoenix roebelenii* (Arecaceae; solitary palm)	Japan	[101]
			*Satakentia liukiuensis* (Arecaceae; solitary palm)	Japan	[101]
			*Syagrus coronata* (Arecaceae; solitary palm)	Brazil	[131]
			*Syagrus romanzoffiana* (Arecaceae; solitary palm)	Argentina	[130]
			*Trachycarpus fortunei* (Arecaceae; solitary palm)	Japan	[101]
			*Washingtonia robusta* (Arecaceae; solitary palm)	Japan	[101]
7	*E. thwaitesii* (Type species)	*Cissus oreophila* (Vitaceae)		Ghana	[132]
		*Cissus* sp. (Vitaceae)		Ghana	[133]
				Sri Lanka	[133]
			*Oncosperma* sp. (Arecaceae; clustering, rarely solitary palm)	Ghana	[133]
				Sri Lanka (Holotype)	[133]

* Have molecular data.

## Data Availability

Not applicable.
